# Rational Design of MOF‐Based Multifunctional Bio‐Nanoreactor for Efficient Detection and Photo‐Degradation of Chloramphenicol

**DOI:** 10.1002/advs.202414866

**Published:** 2025-05-14

**Authors:** Lu Ran, Niu Feng, Yiming Dong, Huanyu Cai, Yiping Chen, Huailong Teng

**Affiliations:** ^1^ College of Chemistry Huazhong Agricultural University Wuhan 430070 China; ^2^ Hubei Key Laboratory of Natural Products Research and Development College of Biological and Pharmaceutical Sciences China Three Gorges University Yichang 443002 China; ^3^ College of food science and technology Huazhong Agricultural University Wuhan 430070 China; ^4^ School of food science and technology Dalian Polytechnic University Dalian 116034 China

**Keywords:** chloramphenicol, detection, metal–organic frameworks, multifunctional bio‐nanoreactor, photo degradation

## Abstract

Food safety have received increasing attention in recent years, and rapid detection and thorough removal of organic contaminants is an important part of food safety control. In this work, a novel multi‐functional photo‐enzymatic nanoreactor HRP@Fe‐NU‐1003 is developed through the co‐immobilization of horseradish peroxidase (HRP) and FeCl_2_ on a photosensitive metal–organic frameworks (MOF) NU‐1003. The bio‐nanocluster can serve as an efficient biosensor in the detection of chloramphenicol (CAP), with a detection limit of 15.38 pg mL^−1^, which is 62 times greater than that of the conventional HRP‐ enzyme‐linked immunosorbent assay method. Besides its detecting capability, the nanoreactor also exhibits high efficiency in the photocatalytic degradation of CAP, which can remove 50 µg mL^−1^ of CAP thoroughly within 30 min, and the mineralization efficiency of CAP reaches 61%. In this material, Fe‐NU‐1003 not only acts as a protecting shell to prevent HRP from deactivation, but improves detecting sensitivity and photocatalytic performance. Mechanism studies show that FeCl_2_ enhances its photocatalytic performance through promoting electron (e^−^)–hole (h^+^) separation and photocurrent transfer. More importantly, the heterogeneous material possesses high stability and can be recycled at least five rounds while its photocatalytic performance maintained at a high level. This strategy provides a new approach for the detection and degradation of pollutants.

## Graphical abstract

A multi‐functional photo‐enzymatic nanoreactor HRP@Fe‐NU‐1003 is developed through co‐immobilization of horseradish peroxidase and FeCl_2_ on a photosensitive metal–organic frameworks NU‐1003. This material showcases remarkable sensitivity and efficacy in both detecting and subsequently photo‐degrading chloramphenicol residues in food products.

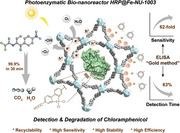

## 1 Introduction

In recent years, the severity of food safety issues has escalated, contaminants such as antibiotics,^[^ ^1^, ^2^, ^3^ ^]^ foodborne pathogens,^[^ ^4^, ^5^ ^]^ mycotoxins,^[^ ^6^, ^7^ ^]^ heavy metals,^[^ ^8^, ^9^ ^]^ pesticide residues,^[^ ^10^, ^11^ ^]^ and aromatic hydrocarbons^[^ ^12^, ^13^ ^]^ that remain in food posing significant risks to human health. Chloramphenicol (CAP) is a naturally occurring broad‐spectrum antibacterial agent that used to be widely employed for the treatment of bacterial diseases in humans and animals.^[^ ^14^, ^15^, ^16^, ^17^ ^]^ However, the widespread usage of antibiotics has caused significant side effects on humans such as liver injury, anaphylaxis, and nervous system destruction.^[^ ^18^, ^19^, ^20^ ^]^ The rapid detection and thorough removal of CAP are crucial to ensure the safety and quality of our food supply. Predominantly, current organic contaminant detection relies on chromatography techniques and colorimetric immune sensing.^[^ ^21^, ^22^, ^23^, ^24^, ^25^ ^]^ Although chromatography technology exhibits high accuracy, the reliance on specialized instruments and long analysis cycle restricts its widespread application.^[^ ^26^, ^27^ ^]^ In comparison, colorimetric immune sensing method represents a simple and rapid way that has potential for widespread applications once the issues of poor reproducibility, high costs, and the inevitable enzyme deactivation have been overcome.^[^ ^28^ ^]^ On the other hand, food contaminant removal is also a prominent research area, generally encompassing two principal strategies: physical adsorption and chemical degradation with porous materials,^[^ ^29^, ^30^, ^31^ ^]^ among them, chemical degradation can completely degrade pollutants into carbon dioxide (CO_2_) and water (H_2_O),^[^ ^1^, ^18^, ^32^, ^33^, ^34^, ^35^ ^]^ especially photocatalytic degradation has become a research hotspot in recent years.^[^ ^36^ ^]^ Therefore, developing multifunctional nanoreactors possessing both pollutant detection and degradation functions would be of important research significance.

Metal–organic frameworks (MOFs) materials with periodic network structures formed through the metal ions or metal clusters with organic ligands have been widely employed in molecular separation and purification,^[^ ^37^ ^]^ drug delivery,^[^ ^38^, ^39^, ^40^ ^]^ energy storage,^[^ ^41^, ^42^, ^43^, ^44^ ^]^ and photocatalysis.^[^ ^45^, ^46^, ^47^, ^48^, ^49^, ^50^, ^51^ ^]^ Their enhanced porosity and good dispersibility in water offer the potential for enzyme immobilization and contaminant adsorption,^[^ ^52^, ^53^ ^]^ thus providing efficiency for detection and photo‐degradation of food contamination.^[^ ^54^ ^]^ For example, Li reported an effective detection of mycotoxins by a highly luminescent MOF material,^[^ ^55^ ^]^ Liu developed a fluorescence and colorimetric biosensor for organophosphorus pesticides detection through capsulation of AuNCs with aggregation induced emission effect into MOF.^[^ ^56^ ^]^ While Wang developed a novel p–n heterojunction photocatalyst BiOI/UiO‐66 for efficient sulfadiazine elimination,^[^ ^57^ ^]^ Mahjoub prepared g‐C_3_N_4_@H/SMOF NCs for visible light photocatalytic 4‐nitrophenol degradation.^[^ ^58^ ^]^ However, only a few composite materials that have both detection and degradation functions have been reported to date, Singhal realized the concomitant photocatalytic removal and real‐time monitoring of noxious fluoroquinolones with a well‐designed Z‐scheme g‐C_3_N_4_/ZnO/NiFe_2_O_4_ heterostructure.^[^ ^59^ ^]^ Besides, it is well known that Fe‐based catalysts could promote the generation of free radicals in photocatalytic processes thus improving the detecting sensitivity and photodegradation ability of composite materials.^[^ ^60^, ^61^, ^62^, ^63^ ^]^ In principle, a rationally designed MOF‐based photoenzymatic nanocluster could kill many birds with one stone, unfortunately, there has been no relevant work reports heretofore.

**Figure 1:**
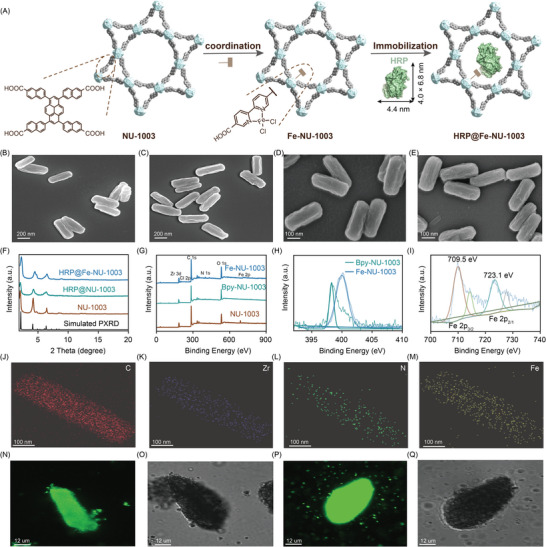
A) Schematic diagram of HRP@Fe‐NU‐1003; SEM images of the B) NU‐1003, C) Fe‐NU‐1003, D) HRP@NU‐1003, E) HRP@Fe‐NU‐1003; F) PXRD patterns; G) XPS spectra NU‐1003, Bpy‐NU‐1003, and Fe‐NU‐1003; H) N 1s XPS spectra of Fe‐NU‐1003 and Bpy‐NU‐1003; I) Fe 2p XPS of Fe‐NU‐1003; TEM‐EDS mapping images of Fe‐NU‐1003 showing the distributions of the elements J) C, K) Zr, L), N, and M) Fe; CLSM images of N,O) HRP@NU‐1003, P,Q) HRP@Fe‐NU‐1003.

Herein, we reported a novel multifunctional photoenzymatic nano‐bioreactor HRP@Fe‐NU‐1003 through the immobilization of horseradish peroxidase (HRP) and (Bpy)FeCl_2_ on photosensitive NU‐1003 material (**Figure** **1**A). This bio‐composite material has demonstrated good sensitivity (15.38 pg mL^−1^) and repeatability in the detection of chloramphenicol in urine, fish, and wastewater, while efficiently adsorbing and degrading chloramphenicol. Also, its high stability allows it to maintain good performance even when recycled for five times. The well‐designed Fe‐NU‐1003 not only acts as a safety cover to prevent HRP from deactivation but also performs as a photocatalyst for light degradation of chloramphenicol. Optoelectronic performance characterization and DFT calculation revealed that (Bpy)FeCl_2_ units in Fe‐NU‐1003 play a vital role in this photoenzymatic bio‐nanoreactor which strengthens its photo‐redox properties through promoting the separation of photoelectrons (e^−^) and holes (h^+^).

## 2 Results and Discussion

According to the solvothermal method reported in the literature,^[^ ^64^, ^65^, ^66^ ^]^ NU‐1003 was synthesized and characterized by Scanning Electron Microscope (SEM) and Transmission Electron Microscope (TEM). As depicted in Figure 1B, NU‐1003 exhibited a consistent and orderly hexagonal cylindrical morphology. Subsequently, bipyridine units were successfully integrated into the Zr metal clusters within NU‐1003 through a solvothermal process, resulting in Bpy‐NU‐1003 which contains metal coordination sites. Then FeCl_2_ was introduced into the MOF framework to give Fe‐NU‐1003. As evidenced in Figure 1C and Figure S1A (Supporting Information), Fe‐NU‐1003 retains a similar morphology to that of NU‐1003, demonstrating that the incorporation of iron does not alter the structural integrity of the material. Importantly, during the coordination process, no iron nanoparticles were formed, the iron element was introduced into the material in the form of metal ions. This ensures a uniform distribution of iron within the MOF framework, providing a stable and well‐defined active site for potential applications.

The powder X‐ray diffraction (PXRD) patterns of the NU‐1003 crystals exhibited a remarkable congruence with the simulated patterns (Figure 1F), confirming the successful preparation of NU‐1003. The elemental compositions and electronic states of Fe‐NU‐1003 were thoroughly analyzed through X‐ray photoelectron spectroscopy (XPS), and the XPS full spectrum revealed the clear presence of Zr, C, O, Cl, N, and Fe, providing definitive evidence for the successful incorporation of iron into the Bpy‐NU‐1003 framework (Figure 1G). Upon comparing the fine spectra of the N elements in Fe‐coordinated Fe‐NU‐1003 with those of the non‐coordinated counterpart, a notable shift toward higher binding energy was observed in the N 1s XPS spectrum of Fe‐NU‐1003. This observation indicated the presence of strong interactions between Fe atoms with the N atoms in the bipyridine ligand, the main peaks at 709.5 and 723.1 eV in the spectra of Fe 2p were attributed to 2p_1/2_ and 2p_3/2_ orbital states of Fe^2+^ (Figure 1H,I). Furthermore, the distribution of elements in the material was analyzed by TEM energy dispersive X‐ray spectroscopy (TEM‐EDS), indicated that the loading amount of iron in Fe‐NU‐1003 was ≈3.28 wt%, the TEM‐EDS images also showed a uniform distribution of C, Zr, N, and Fe elements in Fe‐NU‐1003 (Figure 1J–M).

Horseradish peroxidase (HRP), an enzyme with dimensions of ≈4.0 × 4.4 × 6.8 nm^3^, possesses high catalytic activity towards hydrogen peroxide and thus has been widely employed in food pollutant detection.^[^ ^67^, ^68^ ^]^ Through simple physical adsorption, HRP was successfully immobilized onto both NU‐1003 and Fe‐NU‐1003, resulting in HRP@NU‐1003 and HRP@Fe‐NU‐1003 without altering the morphological features of the underlying MOF frameworks (Figure 1D,E). The PXRD pattern of HRP@NU‐1003 and HRP@Fe‐NU‐1003 revealed that the biological clusters did not disrupt the underlying crystal structure of the NU‐1003 MOF framework (Figure 1F). The bicinchoninic acid assay (BCA) provided quantitative evidence that ≈25% and 10% of the HRP was encapsulated within the pore canals of NU‐1003 and Fe‐NU‐1003, respectively. The successful immobilization of HRP onto the MOFs was further confirmed by Fourier Transform Infrared Spectroscopy (FT‐IR) analysis (Figure S1B, Supporting Information). Moreover, the distribution of HRP within the bioclusters was detected, HRP was labeled with fluorescein isothiocyanate (FITC) probe and then immobilized by NU‐1003 and Fe‐NU‐1003 to give labeled HRP^*^@NU‐1003 and HRP^*^@Fe‐NU‐1003. Confocal laser scanning microscopy (CLSM) images suggested that FITC‐enzyme (green) was evenly dispersed within the internal pores of NU‐1003 and Fe‐NU‐1003 (Figure 1N–Q).

With these biological materials in hand, we first investigated the catalytic performance of four materials, including HRP@NU‐1003, HRP@Fe‐NU‐1003, NU‐1003, and Fe‐NU‐1003 in hydrogen peroxide decomposition (Figure S2, Supporting Information). Given the significantly superior catalytic performance of HRP@Fe‐NU‐1003 among the four materials under comparison, it was selected as signal probe for the development of biosensor.

**Figure 2:**
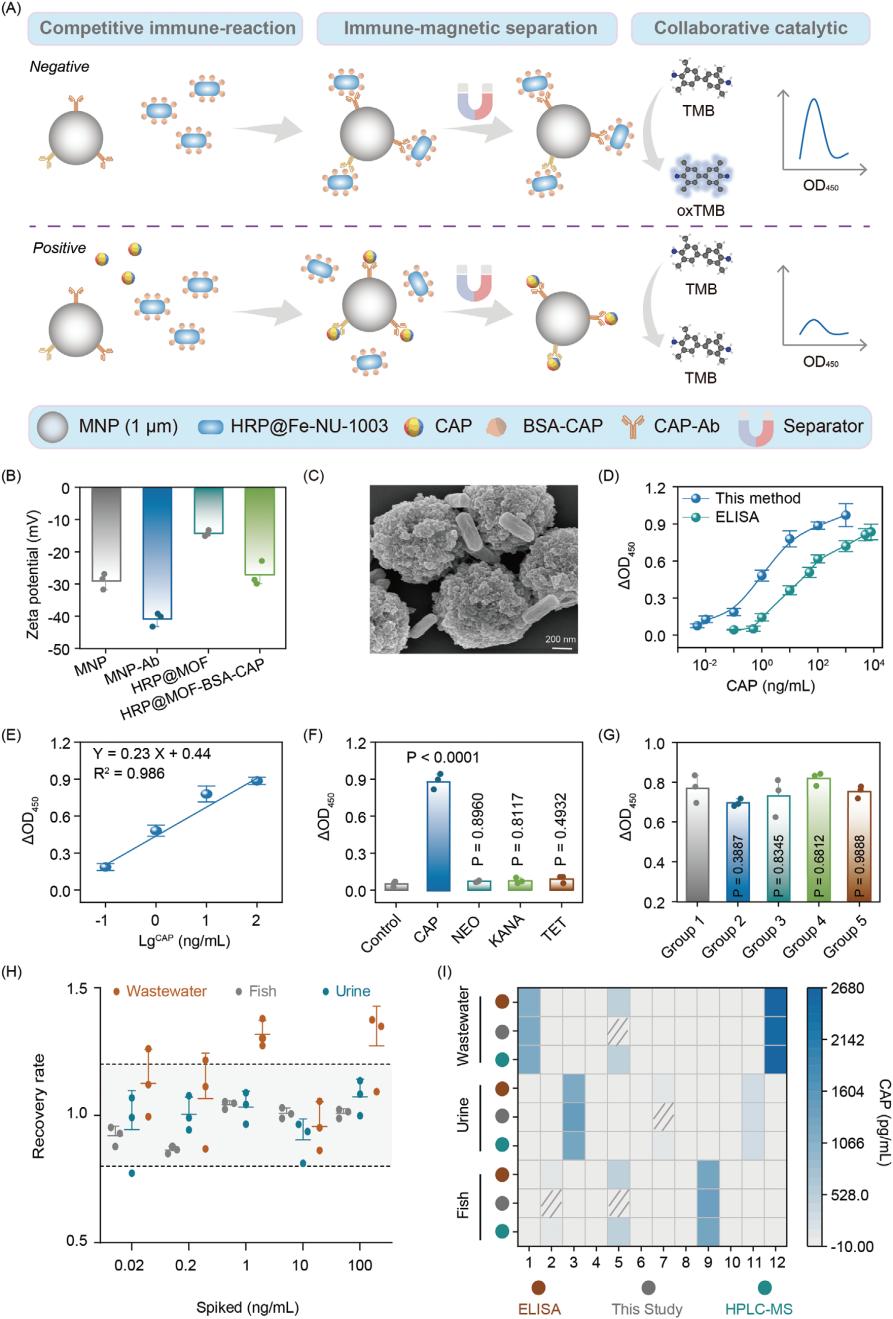
Detection of CAP using the HRP@Fe‐NU‐1003‐based biosensor. A) Schematic diagram of the detection of CAP; B) Zeta potential of MNP and HRP@Fe‐NU‐1003 (HRP@MOF) before and after coupling with CAP‐Ab and BSA‐CAP; C) SEM of the complex for MNP‐CAP‐Ab, CAP, and HRP@Fe‐NU‐1003‐BSA‐CAP; D) Standard curve, E) linear range, F) specificity tests, G) anti‐interference capability (Group 1: CAP, Group 2: mixed CAP and NEO, Group 3: mixed CAP and KANA, Group 4: mixed CAP and TET, Group 5: mixed CAP, NEO, KANA and TET), H) spiked recovery rate and I) real sample testing for CAP detection. p‐values were calculated using two‐sided One‐way ANOVA post‐Dunnett's test. The error bars represent mean ± SD from triplicate replications (n = 3).

The detection principle of the biosensor is illustrated in **Figure** **2**A. In this system, magnetic nanoparticles (MNPs) and HRP@Fe‐NU‐1003 surfaces were functionalized with biometric molecules, serving as capture and detection probes, respectively. When the sample containing the target analyte such as CAP, a significant fraction of the antibody sites on the HRP@Fe‐NU‐1003 surface was preferentially occupied by CAP. The amount of CAP directly determines the number of complexes formed between the capture and detection probes. Thus, the CAP content can be indirectly quantified by measuring the number of these complexes. Meanwhile, the magnetic nature of the MNPs facilitates efficient separation and concentration of the analyte‐bound probes, thereby enhancing the sensitivity and specificity of the detection process.

After coupling with biological recognition molecules, both the magnetic nanoparticles (MNPs) and HRP@Fe‐NU‐1003 experienced a notable increase in hydration particle size, as depicted in Figure S3A (Supporting Information). This expansion can be attributed to the added layer of proteins and other molecules attached to their surfaces. Concurrently, a more negative zeta potential was observed, with MNPs exhibiting a decrease of 11.80 mV and HRP@Fe‐NU‐1003 demonstrating a decrease of 12.89 mV (Figure 2B). This may be due to the abundance of negatively charged groups present in the proteins. Additionally, the characterization of these nanoparticles through various techniques further validates their successful modification and the changes in their physicochemical properties upon coupling with biological recognition molecules (Figure 2C; Figure S3B, Supporting Information).

To optimize the detection performance of the biosensor, the dosage of the coupling agent and biometric molecules used to functionalize the nanoparticles, the ratio between the capture probe and the detection probe, and the duration of the competitive reaction were investigated (Figure S4, Supporting Information). Indeed, after evaluating the various factors that impact detection performance, it was determined that the improved analytical results observed with longer reaction times could be attributed to the limited availability of binding sites following the achievement of immune reaction equilibrium. Consequently, a dosage of 50 µg mL^−1^ of MNP‐Ab (magnetic nanoparticles functionalized with antibodies) and 125 µg mL^−1^ of BSA‐CAP@HRP@Fe‐NU‐1003 (CAP‐bovine serum albumin conjugate was covalently coupled to the surface of Fe‐NU‐1003 which incorporating HRP), along with a competitive reaction time of 15 min, to achieve optimal biosensor performance. A linear detection range of 100 pg mL^−1^–100 ng mL^−1^ (Y = 0.23 X + 0.44, R^2^ = 0.986), along with a limit of detection (LOD) of 15.38 pg mL^−1^ was obtained under optimal conditions (S = 0.0048, M = 0.936) (Figure 2D,E). The HRP@Fe‐NU‐1003‐based biosensor surpasses traditional enzyme‐linked immunosorbent assays (ELISA) in terms of sensitivity. The improvement in sensitivity over ELISA is attributed to two key factors: i) The dual catalytic signal amplification strategy, which involves the synergistic use of Fe^2+^ and HRP within the HRP@Fe‐NU‐1003 probe. This approach amplifies the signal generated during the detection process, leading to a more sensitive response; ii) The uniform size of the HRP@Fe‐NU‐1003 nanoparticles, coupled with their smooth surface and good dispersibility, play a crucial role in improving the sensitivity of the biosensor. These characteristics contribute to optimal conditions for nanoparticle collisions during the immune reaction process. A comprehensive comparison of the HRP@Fe‐NU‐1003‐based biosensor with reported methods is provided in Table S1 (Supporting Information).

Not only that, the HRP@Fe‐NU‐1003‐based biosensor also exhibits good selectivity and specificity in targeted interfering substances, including neomycin (NEO), kanamycin (KANA), and tetracycline (TET). The results in Figure 2F,G demonstrated that only the presence of CAP induced significant absorbance changes, in contrast, other substances tested in the experiments exhibited negligible effects on the absorbance. The real‐world testing of CAP in wastewater, fish, and urine has further validated the excellent specificity and reliability of the HRP@Fe‐NU‐1003‐based biosensor. The high spiked recoveries ranging from 86.39% to 131.82%, combined with low relative standard deviations (RSD) between 1.51% and 16.72%, demonstrate the biosensor's ability to accurately detect CAP in complex systems (Figure 2H; Table S2, Supporting Information). Additionally, the analytical performance of the biosensor in real samples has shown good alignment with results obtained from established methods such as high‐performance liquid chromatography‐mass spectrometry (HPLC‐MS) and ELISA (Figure 2I; Table S3, Supporting Information). In the case of a low‐concentration specimen with residual CAP, both HPLC‐MS and the biosensor provided positive results, while ELISA failed. This discrepancy underscores the higher sensitivity of the biosensor and its ability to accurately detect even trace amounts of analytes. Overall, the HRP@Fe‐NU‐1003‐based strategy offers significant advantages over traditional ELISA methods in terms of detection time and sensitivity. By achieving a 63% reduction in detection time and a 62‐fold increase in sensitivity, the biosensor demonstrates its ability to provide faster and more accurate detection of analytes.

**Figure 3:**
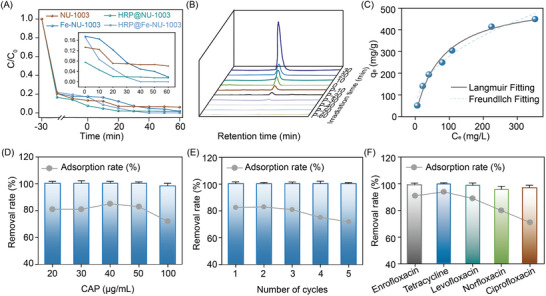
A) Kinetics of adsorption and photocatalytic degradation of CAP by different samples. B) HPLC chromatograms of CAP adsorption and degradation. C) Adsorption isotherms of CAP onto HRP@Fe‐NU‐1003, and non‐linear fits of Langmuir and Freundlich models. D) Effect of CAP dosage. E) Cyclic of adsorption and photocatalytic degradation experiments by HRP@Fe‐NU‐1003. F) Adsorption and photocatalytic degradation of diverse antibiotics by HRP@Fe‐NU‐1003.

In addition to chloramphenicol detection, the newly designed bio‐nanocluster was next attempted for the adsorption and photo‐degradation of CAP. The results presented in **Figure** **3**A indicated that the HRP@Fe‐NU‐1003 nanomaterial showed the highest performance in both adsorption and photocatalytic experiments, outperforming the other tested porous nanomaterials including HRP@NU‐1003, Fe‐NU‐1003, and NU‐1003. Meanwhile, the mineralization rate of CAP was 61%. To demonstrate the importance of light and HRP@Fe‐NU‐1003 in this process. A series of control experiments were conducted. The results of Figure S5 (Supporting Information) indicated that the removal rate of CAP was 0% when there was no material under illumination. In addition, the adsorption and photo‐degradation of CAP cannot be carried out with only hydrogen peroxide (H_2_O_2_). These results indicated that HRP@Fe‐NU‐1003 was indispensable. As depicted in Figure 3B, the HPLC chromatogram revealed that complete degradation of chloramphenicol (CAP) was achieved within just 30 min of light irradiation. Under light conditions, the HRP@Fe‐NU‐1003 composite material exhibits a remarkable dual degradation function. Not only does it harness the Fenton reaction, catalyzed by Fe^2+^ ions, to decompose hydrogen peroxide into hydroxyl radicals for CAP degradation, but it also leverages the horseradish peroxidase (HRP) to oxidize hydrogen peroxide, generating additional free radicals that contribute to the photo‐degradation process. This dual function ensures a heightened and efficient photocatalytic degradation of CAP, showcasing the composite material's versatility and potential in environmental remediation applications.

Furthermore, adsorption isotherm models provide valuable insights into the interaction between adsorbents and adsorbate molecule. In this case, two prominent models, the Langmuir and Freundlich isotherms, were employed to fit the adsorption behavior of CAP on the HRP@Fe‐NU‐1003 surface (Figure 3C). The R^2^ value suggested that the Langmuir model provided a better representation of the adsorption isotherm of CAP on the HRP@Fe‐NU‐1003 composite compared to the Freundlich model (Table S4, Supporting Information). This finding indicates that the adsorption process of CAP onto the surface of HRP@Fe‐NU‐1003 primarily follows a monolayer adsorption mechanism.

The investigation into the adsorption and degradation capabilities of HRP@Fe‐NU‐1003 across various concentrations of chloramphenicol (CAP) highlights showed remarkable removal rates of 97–100% observed over the concentration range of 20–100 µg mL^−1^, demonstrating its capability to effectively address CAP contamination, even at relatively high concentrations. Moreover, HRP@Fe‐NU‐1003 exhibited only a slight decrease in the removal rate for CAP even after five consecutive adsorption‐photocatalytic degradation cycles (Figure 3E), while the recycled material possessed same structural and morphological integrity as original material (Figure S5, Supporting Information). Moreover, only 0.05 wt.% of Fe leaching was observed after the five cycle. In addition, this material possesses a broad spectrum of activity against different types of antibiotic contaminants in water, including enrofloxacin, tetracycline, levofloxacin, norfloxacin, and ciprofloxacin (Figure 3F).

**Figure 4:**
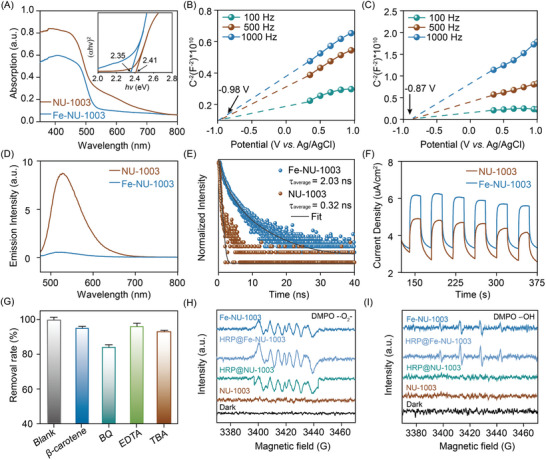
A) UV/vis diffuse reflectance spectra of NU‐1003 and Fe‐NU‐1003. B) Mott–Schottky plots of Fe‐NU‐1003 C) and NU‐1003. D) Steady‐state photoluminescence spectra of NU‐1003 and Fe‐NU‐1003 excited by a 450 nm laser. E) Excited state lifetime measurement through TCSPC. F) Photocurrent response of NU‐1003 and Fe‐NU‐1003. G) Comparison of the removal rate under different scavengers. EPR spectra of ·O_2_^−^ H) and ·OH I). Error bars indicate the standard deviation of three measurements of quenching experiment.

To gain a profound understanding of the exceptional detecting and photocatalytic performance of this material, we delved into the photoelectrochemical properties brought about by the incorporation of metal ions. Specifically, we examined how these ions alter the fundamental optical and electronic attributes of the material, ultimately enhancing its functionality. The light absorption characteristics of materials are inherently connected to their electronic excitation capabilities. Thus, to assess the band gap and absorption edge of the synthesized NU‐1003 and Fe‐doped Fe‐NU‐1003, UV/Vis diffuse reflectance spectra (DRS) measurements were conducted. As shown in **Figure** **4**A, the absorption edge of the undoped NU‐1003 was observed to lie approximately at 524 nm. In contrast, the introduction of Fe ions into the structure of NU‐1003, resulting in Fe‐NU‐1003, shifted the absorption edge to 543 nm. The optical bandgap was calculated to the following literature method.^[^ ^69^, ^70^ ^]^ The pristine NU‐1003 was calculated to be 2.41 eV using the Tauc plot method, whereas the optical bandgap of Fe‐NU‐1003 narrowed to 2.35 eV. The precise positioning of the conduction band (CB) and valence band (VB) energy levels is equally crucial in determining the photocatalytic prowess of a material. To gain insight into these energy levels, the flat band potential (*E*_fb_) was experimentally determined using Mott‐Schottky (MS) analysis. The results indicated that the *E*_fb_ of the pristine NU‐1003 was −0.87 V (vs Ag/AgCl), whereas the Fe‐doped Fe‐NU‐1003 exhibited a more negative *E*_fb_ of −0.98 V (vs Ag/AgCl) (Figure 4B,C). According to the energy band equation (*E*_g_ = *E*_VB_–*E*_CB_), the VB position of NU‐1003 and Fe‐NU‐1003 were calculated to be 1.74 and 1.57 V (vs NHE), respectively (Figure S6, Supporting Information). Furthermore, the results presented in Figure 4D, showing the steady‐state photoluminescence (PL) spectra at an emission wavelength of 450 nm, provide valuable insights into the migration and separation efficiency of photo‐induced carriers in both NU‐1003 and Fe‐NU‐1003. The observation that Fe‐NU‐1003 exhibits a lower PL intensity compared to NU‐1003 is a strong indication that the recombination of photo‐excited electron‐hole pairs is indeed significantly suppressed in the Fe‐doped material. The excited‐state lifetimes of NU‐1003 and Fe‐NU‐1003 were measured by using time‐correlated single photon counting (TCSPC), revealing the average lifetime of NU‐1003 and Fe‐NU‐1003 were calculated to be 0.32 and 2.03 ns, respectively (Figure 4E). The observation of a significantly longer excited‐state lifetime for Fe‐NU‐1003 compared to its undoped counterpart NU‐1003. Besides, photocurrent (Figure 4F) and electrochemical impedance experiments (Figure S7, Supporting Information) provide further evidence that Fe‐NU‐1003 exhibits improved electron transfer capabilities compared to its undoped counterpart NU‐1003. These results strongly support the notion that the introduction of Fe^2+^ ions enhance the photogenerated carrier migration efficiency.

The generation of reactive oxygen species (ROS) holds paramount importance in the photocatalytic degradation of chloramphenicol. Thereby, in order to determine the primary reactive oxygen species (ROS) formed during CAP degradation, free radical quenching experiments were conducted. As depicted in Figure 4G, a significant reduction of CAP removal was observed in the presence of BQ and TBA, which are known inhibitors of superoxide radicals (∙O_2_^−^) and hydroxyl radicals (∙OH), respectively. This suggested that ∙O_2_^−^ and ∙OH were predominant ROS in the photo‐degradation process. Furthermore, the contribution rates of different active species were as follows: ∙O_2_^−^ (50%) > ∙OH (22%) > ^1^O_2_ (16%) > h^+^ (12%) (Figure S8, Supporting Information). To further confirm the production of ∙O_2_^−^ and ∙OH during photocatalysis, electron paramagnetic resonance (EPR) spectroscopy was employed. Figure 4H illustrated that notable EPR signals for ∙O_2_^−^ were detected from Fe‐NU‐1003, HRP@Fe‐NU‐1003, and HRP@NU‐1003 under light exposure, with HRP@Fe‐NU‐1003 exhibiting the strongest signal. In contrast, Figure 4I shows that ∙OH radicals were only detected in the presence of Fe‐NU‐1003 and HRP@Fe‐NU‐1003, while NU‐1003 and HRP@NU‐1003 failed to produce ∙OH. These results underscore the critical role of ∙O_2_^−^ in the photodegradation of CAP, with ∙OH playing a supportive role. As known, ∙O_2_^−^ and ∙OH are generated by the Fenton reaction, and the H_2_O_2_ is one of the important reactants. Therefore, the concentration of H_2_O_2_ was detected during the photocatalytic process. As shown in Table S5 (Supporting Information), the concentration of H_2_O_2_ has significantly increased with the reaction time increasing. The concentration of H_2_O_2_ reached to 17.1 µg mL^−1^ at 30 min of the reaction process.

**Figure 5:**
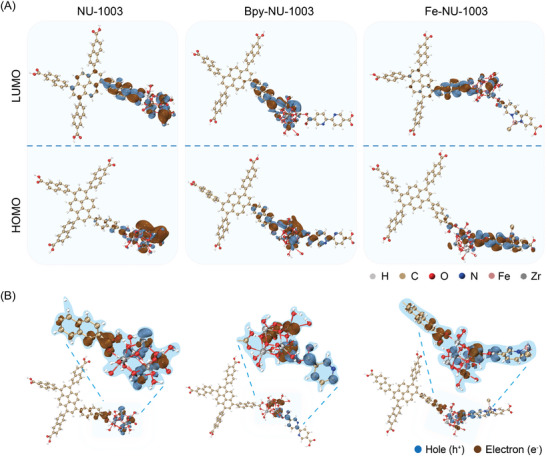
A) LUMO and HOMO orbitals distribution in NU‐1003, Bpy‐NU‐1003, and Fe‐NU‐1003 from DFT calculation. B) Electron (brown) and hole (blue) distribution in NU‐1003, Bpy‐NU‐1003, and Fe‐NU‐1003.

Furthermore, to gain deeper insights into the electronic structure and charge distribution within the metal–organic frameworks (MOFs) NU‐1003 and Fe‐NU‐1003, time‐dependent density functional theory (TDDFT) calculations were performed to determine their highest occupied molecular orbital (HOMO) and lowest unoccupied molecular orbital (LUMO) energies. These calculations provide valuable information on the reactivity and charge transfer properties of the MOFs. As illustrated in **Figure** **5**A, the HOMO of NU‐1003 was mostly localized on the Zr_6_ clusters, while the LUMO was distributed across the naphthyl parts and Zr_6_ clusters. Meanwhile, the introduction of bipyridine does not significantly alter the distribution of the LUMO and HOMO.

The introduction of metal ions has significantly altered the landscape of the LUMO and HOMO within the material. These orbitals are now predominantly found in the regions associated with bipyridine‐Fe and Zr_6_ clusters. This redistribution of the LUMO and HOMO is crucial as it effectively inhibits the recombination of photoelectrons and holes, a process that is key to enhancing the photocatalytic efficiency of the material. Additionally, Figure 5B illustrates the theoretical distribution of photoelectrons (e^−^) and holes (h^+^) within the MOF materials. It is observable that there is an overlap between the regions populated by electrons and holes in NU‐1003. Specifically, the holes are primarily distributed across the pyrene units, while the electrons are predominantly situated at the Zr_6_ clusters. Upon the introduction of bipyridine to the MOF material, the distribution of photoelectrons (e^−^) and holes (h^+^) shifts. The photoelectrons and holes now reside primarily at the Zr_6_ clusters and bipyridine sites, respectively. However, in Fe‐NU‐1003, there is a nearly complete spatial separation of electrons and holes, the holes in Fe‐NU‐1003 are mainly distributed on the pyrene units, while the electrons are localized on the bipyridine‐Fe complexes, which is a crucial factor for enhanced photocatalytic performance. The inhibition of recombination in Fe‐NU‐1003 is further evidence by the observed higher photocatalytic activity, making it a more effective photocatalyst compared to its counterpart without the bipyridine‐Fe modification.

## 3 Conclusion

In summary, we have devised a versatile nanoreactor that integrates detection and degradation functionalities. This innovative material has demonstrated outstanding performance in detecting chloramphenicol in food samples. Compared with the traditional ELISA, the sensitivity of detection enhances 62 times and the low limit of detection is 15.38 pg mL^−1^. Furthermore, its photocatalytic capabilities allow it to remove chloramphenicol pollutants present in food, utilizing oxygen under visible light conditions. The 50 µg mL^−1^ of CAP is completely removed within 30 min. Moreover, the structural characterization of the nanoreactor material is thoroughly elucidated, and photoelectric property testing and theoretical calculations have unveiled the intricate structure‐performance relationship. The multi‐functional photo‐enzymatic nanoreactor has great potential in addressing food safety issues and reducing antibiotic residues.

## 4 Experimental Section

### Synthesis of NU‐1003

Benzoic acid (1.25 g, 10.25 mmol), ZrOCl_2_·8H_2_O (100 mg, 0.31 mmol), and 25 mL of DMF were added into 50 mL container. The solution was heated at 80 °C for 1 h. After cooling to room temperature, Section S2 (Supporting Information) and trifluoroacetic acid (100 µL) was added into the solution. The mixture was sonicated for 5 min and heated at 120 °C for 3 h. After completing the reaction, the supernatant was filtered. The solid was washed with DMF and acetone. NU‐1003 was activated at 50 °C for 6 h. The product was obtained as a yellow solid.

### Synthesis of Fe‐NU‐1003

NU‐1003 (40 mg), 2,2′‐Bipyridine‐5,5′‐dicarboxylic acid (20 mg), and 40 mL of DMF were added into 50 mL container. The mixture was heated at 80 °C for 24 h. After completing the reaction, the yellow precipitate was filtered and washed with DMF. Then, FeCl_2_ and anhydrous methanol (2 mL) were added into the yellow precipitate under N_2_ atmosphere. The reaction mixture was stirred at room temperature for 12 h. The product was obtained by centrifugal.

## Conflict of Interest

The authors declare no conflict of interest.

## Supporting information



Supporting Information

## Data Availability

The data that support the findings of this study are available in the supplementary material of this article.
