# Supplementary material for: Rational Design of MOF‐Based Multifunctional Bio‐Nanoreactor for Efficient Detection and Photo‐Degradation of Chloramphenicol
Source: Adv Sci (Weinh). 2025 May 14;12(22):2414866. doi: 10.1002/advs.202414866 (PMC12165110; doi:10.1002/advs.202414866)
Supplement: Supplementary file 1 — Supporting Information [file ADVS-12-2414866-s001.docx]

Supporting information

**Rational Design of MOF-based Multifunctional Bio-Nanoreactor for** **Efficient Detection and Photo-degradation of Chloramphenicol**

Lu Ran^[a,d]†^, Niu Feng^[c]†^, Yiming Dong^[c]^, Huanyu Cai^[a]^, Yiping Chen^[b]*^ and Huailong Teng^[a]*^

^a^ College of Chemistry, Huazhong Agricultural University, Wuhan, 430070, China.

^b^ School of food science and technology, Dalian Polytechnic University, Dalian, 116034, China.

^c^ College of Food science and technology, Huazhong Agricultural University, Wuhan, 430070, China.

^d^ Hubei Key Laboratory of Natural Products Research and Development, College of Biological and

Pharmaceutical Sciences, China Three Gorges University, Yichang 443002, China

*Corresponding author

E-mail: [chenyp@dlpu.edu.cn](mailto:chenyp@dlpu.edu.cn) (Y. Chen); [thlong@mail.hzau.edu.cn](mailto:thlong@mail.hzau.edu.cn) (H. Teng)

^†^These authors contributed equally to this work.

Table of Contents

[1. Materials and methods 3](#_Toc179206026)

[2. Synthetic of building blocks 4](#_Toc179206027)

[3. Detection of CAP 5](#_Toc179206028)

[4. Adsorption and photodegradation of CAP 5](#_Toc179206029)

[5. DFT calculations 6](#_Toc179206030)

[6. Supplementary Figures 7](#_Toc179206031)

[7. Supplementary Tables 11](#_Toc179206032)

[8. References 13](#_Toc179206033)

1. Materials and methods

**General information.** All chemical reagents were purchased from Bide pharmtech Ltd or Leyan Chemical Reagent Company, and were directly used without further purification. All solvents were selected as analytical grade without further purification. Pure water was used throughout the entire experimental process.

**Characterization.** Powder X-ray diffraction (PXRD) was measured by Bruker D8 Advance operated at 40 kV and 40 mA with Cu Kα radiation. Fourier transform infrared spectra (FT-IR) were collected on Nicolet iS50. UV-vis diffuse reflectance spectra (DRS) were measured on a Shimadzu UV-3600 Plus UV-vis-NIR spectrophotometer. Steady-state PL spectra (excitation at 330 nm) were recorded on a Shimadzu RF-6000. The scanning electron microscopy (SEM, ZEISS GeminiSEM 300) and transmission electron microscopy (TEM, JEOL JEM-F200) were used to characterize the morphologies of material.

**Electrochemical measurements.** Photoelectrochemical measurements were performed on a CHI660E electrochemical workstation (Chenhua Instruments, China) in a typical three electrode cell. The three electrode cell included Pt plate as the counter electrode, Ag/AgCl as the reference electrode, and a working electrode. The work electrode was prepared as follows: 10 mg of samples were added into a mixed solution of 10 uL Nafion and 200 µL isopropanol, then the resulting suspension was carefully dropped on the ITO glass substrate (10 × 15 × 1.1 mm) and dried overnight at room temperature. 0.1 M Na_2_SO_4_ aqueous solution was used as the electrolyte. The photocurrent measurements were recorded with the light on and off conditions (300 W Xe-lamp, λ ≥ 420 nm). The electrochemical impedance spectroscopy (EIS) measurements used 0.1 M KCl and 0.005 M K_3_[Fe(CN)_6_] aqueous solution as the electrolyte. For Mott-Schottky measurements, the amplitude was 0.005 V with frequencies of 100, 500, and 1000 Hz.

2. Synthetic of building blocks

The 1,3,6,8-Tetra(6-methoxycarbonylnaphthalen-2-yl)pyrene was synthesized according to previous literature reports.^[1]^ Specifically, 1,3,6,8-tetrabromopyrene (1.4 mmol, 0.7 g), Methyl 6-(4,4,5,5-tetraMethyl-1,3,2-dioxaborolan-2-yl)-2-naphthoate (7.0 mmol, 2.2 g), Pd(PPh_3_)_4_ (0.09 mmol, 108.5 mg), and K_3_PO_4_ (25.5 mmol, 5.4 g) were added into a degassed mixture solvent of 1,4-dioxane (60 mL) and H_2_O (2 mL), then the reaction was refluxed at 110 °C for 72 h under N_2_. After cooling to room temperature, water was added to this mixture, the precipitate was filtered and washed with CH_2_Cl_2_. The product was obtained as a yellow power (1.1 g, 80%).

1,3,6,8-Tetra(6-carboxynaphthalen-2-yl)pyrene (1.2 mmol, 1.1 g) was added to a mixed solution of THF (30 mL) and MeOH (15 mL). Then 3 M NaOH solution (7 mL) was added into the above solution. The resulting mixture was refluxed for 24 h. After the reactant was completely consumed, the organic solvent was removed and 100 mL of water was added to dissolve the solid. Concentrated HCl was added to the solution until the PH = 1. The yellow precipitate was filtered and washed with water. The product was purified by recrystallization from DMF. The product was obtained as a yellow power (0.58 g, 55%).

**HRP immobilization in NU-1003 and Fe-NU-1003:** 5 mg NU-1003 was added into the 1 mg/mL HRP solution (10 mM PBS buffer, pH = 7.0, 1 mL), and then NU-1003 was sonicated for 10 min until material dispersion. The mixture was stirred at 4 °C for 24 h. The enzyme content in supernatant was detected with the BCA method and quantified by the standard curve of BSA. Fe-NU-1003 was consistent with the above HRP immobilization method.

**Modification of MNP-COOH and HRP@Fe-NU-1003:** The preparation process of MNP-CAP-Ab and HRP@Fe-NU-1003-BSA-CAP was based on our previous work with some modiﬁcations.^[2]^ Take magnetic nanoparticles as an example: Firstly, 100 μL of MNP (1 μm, 10 mg/mL) were washed with MEST (10 mM MES, pH 6.0, containing 0.05% Tween-20) two times. Secondly, 60 μL of EDC (5 mg/mL) and 30 μL of NHS (5 mg/mL) were add into the tube. Then gently reverse the mixture for 20 min at 37 °C to activate the carboxyl groups on the surface of MNPs. Thirdly, we washed MNP which contain activated carboxyl groups with MEST three times. After that, we added 100 μg of CAP-Ab for the target to the above Eppendorf tube and adjusted the total capacity with PBST (10 mM PBS, pH 7.4, 0.05% Tween-20) to 1 mL and reversed for 3 h at 37 °C. Fourth, the 1 mL of PBST with 1% BSA was used to close the residual sites for 25 min at 37 °C. Lastly, the 1 mL of PBST was used to wash the above conjugate three times; next, we dispersed the MNP-antibody in 1 mL of PBST containing 0.5% BSA and stored it at 4 °C. The modification of the BSA-CAP on the HRP@Fe-NU-1003 was similar to MNP, except that magnetic separation was replaced by centrifugal separation (10000 rpm for 10 min).

3. Detection of CAP

Firstly, the 100 μg/mL of MNP-CAP-Ab, the 2 μg/mL of HRP@Fe-NU-1003-BSA-CAP solution, and the serial concentrations of CAP standard solution were removed from 100 μL respectively to 1.5 mL Eppendorf tube and mildly shaken for 15 min at 37 °C. PBST was washed by magnetic separation. Secondly, 100 μL PBS was used to resuspend the complex and transfer to the ELISA well, then 100 μL TMB was added to the complex solution to react at room temperature for 10 min. Lastly, the colorimetric reaction was terminated by the addition of 2 mM H_2_SO_4_ (50 μL), and the absorbance was measured at 450 nm.

4. Adsorption and photodegradation of CAP

NU-1003, HRP@NU-1003, Fe-NU-1003 and HRP@Fe-NU-1003 were evaluated for the adsorption and photodegradation of CAP in water. The adsorption ability of the MOF materials was explored in a dark environment by adding 5 mg of as-prepared catalyst to 10 mL aqueous solutions containing of 50 μg/mL CAP. The photodegradation of CAP by MOF materials saturated with adsorption was investigated under irradiation with a xenon lamp for 60 min. After the reaction, the concentration of CAP was determined by high performance liquid chromatography (HPLC). The mobile phase of HPLC was acetonitrile-water solution (v/v = 50/50), with the flow rate of 0.8 mL/min and the detection wavelength was 280 nm. Moreover, for the best performing HRP@Fe-NU-1003 was further investigated its adsorption and photodegradation ability for different concentrations of CAP, and the cyclic performance.

5. DFT calculations

All calculations in this work were performed using Gaussian 09 program package^[3]^ Full geometry optimizations were performed to locate all the stationary points, using the B3LYP^[4]^ with the def2svp^[5-6]^ basis, namely B3LYP/def2svp. Dispersion corrections were computed with Grimme's D3(BJ) method in optimization.^[7]^ The excitation energies for singlet and triplet states were calculated by using TD-DFT method at the PBE0/def2svp level^[5,8]^. Harmonic vibrational frequency was performed at the same level to guarantee that there is no imaginary frequency in the molecules, i.e. they locate on the minima of potential energy surface. Convergence parameters of the default threshold were retained (maximum force within 4.5×10^−4^ Hartrees/Bohr and root mean square (RMS) force within 3.0×10^−4^ Hartrees/Radian) to obtain the optimized structure. The optimal structure was identified given that all calculations for structural optimization were successfully converged within the convergence threshold of no imaginary frequency, during the process of vibration analysis.

6. Supplementary Figures


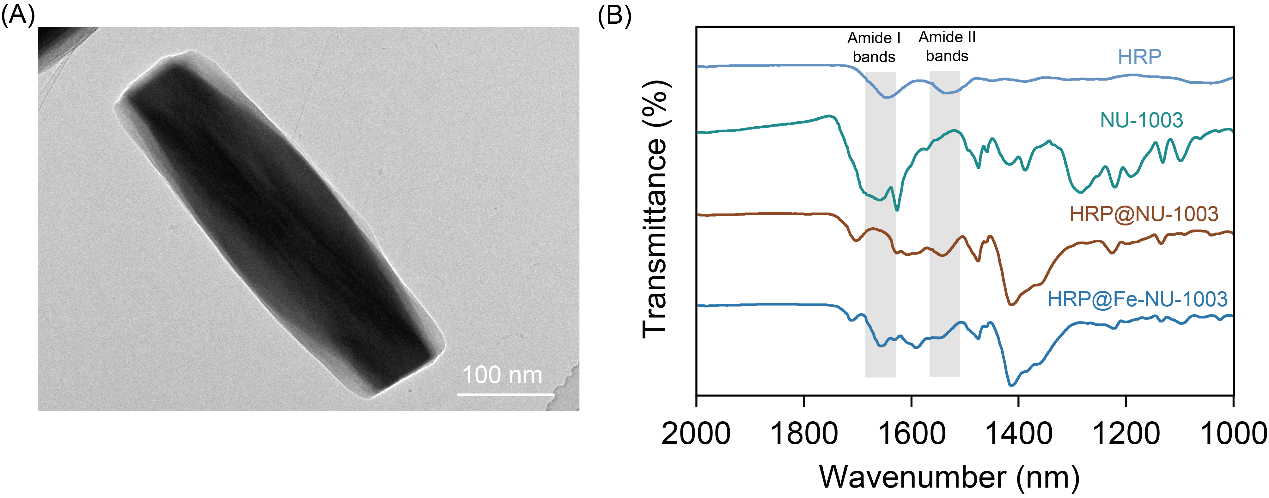


**Figure S1.** (A) TEM images of Fe-NU-1003, (B) FT-IR spectra of different MOF materials and the corresponding enzyme.


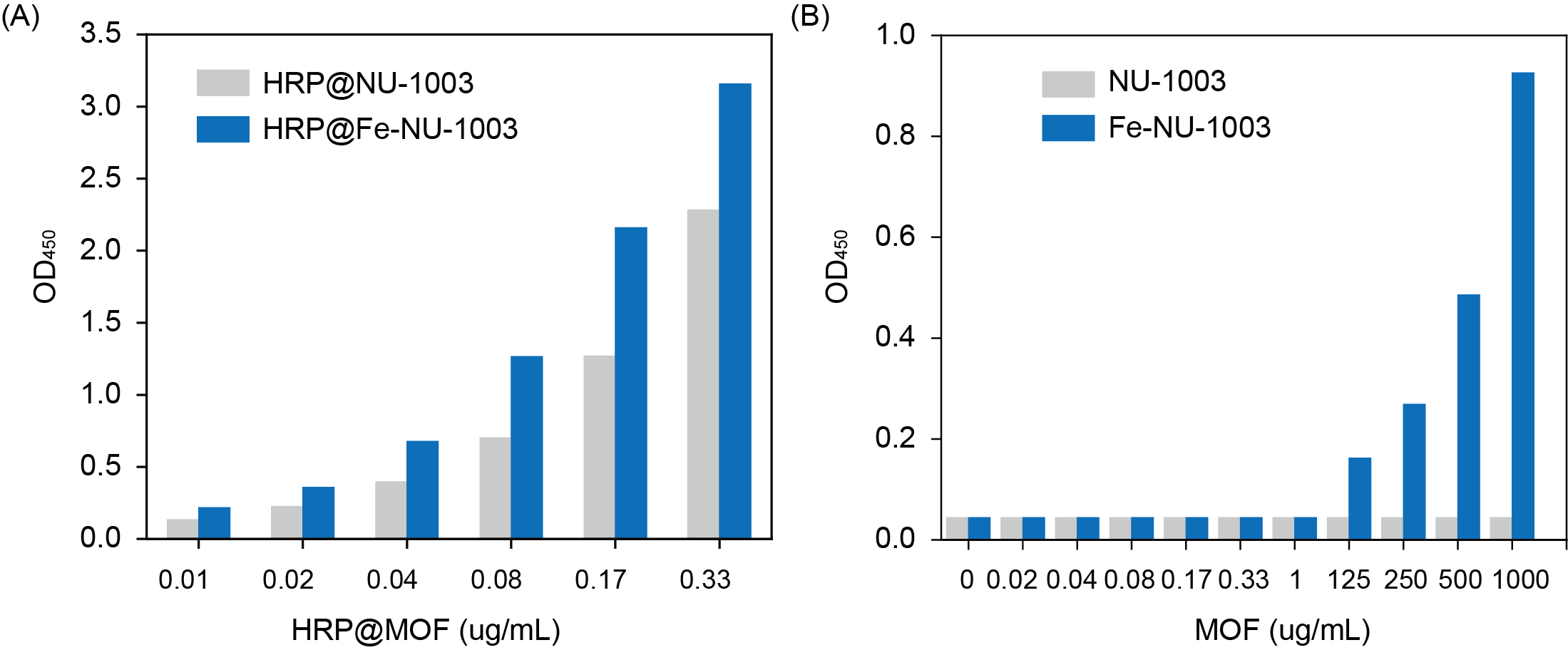


**Figure S2.** TMB color comparison of (A) HRP@NU-1003 and HRP@Fe-NU-1003, (B) NU-1003 and Fe-NU-1003.


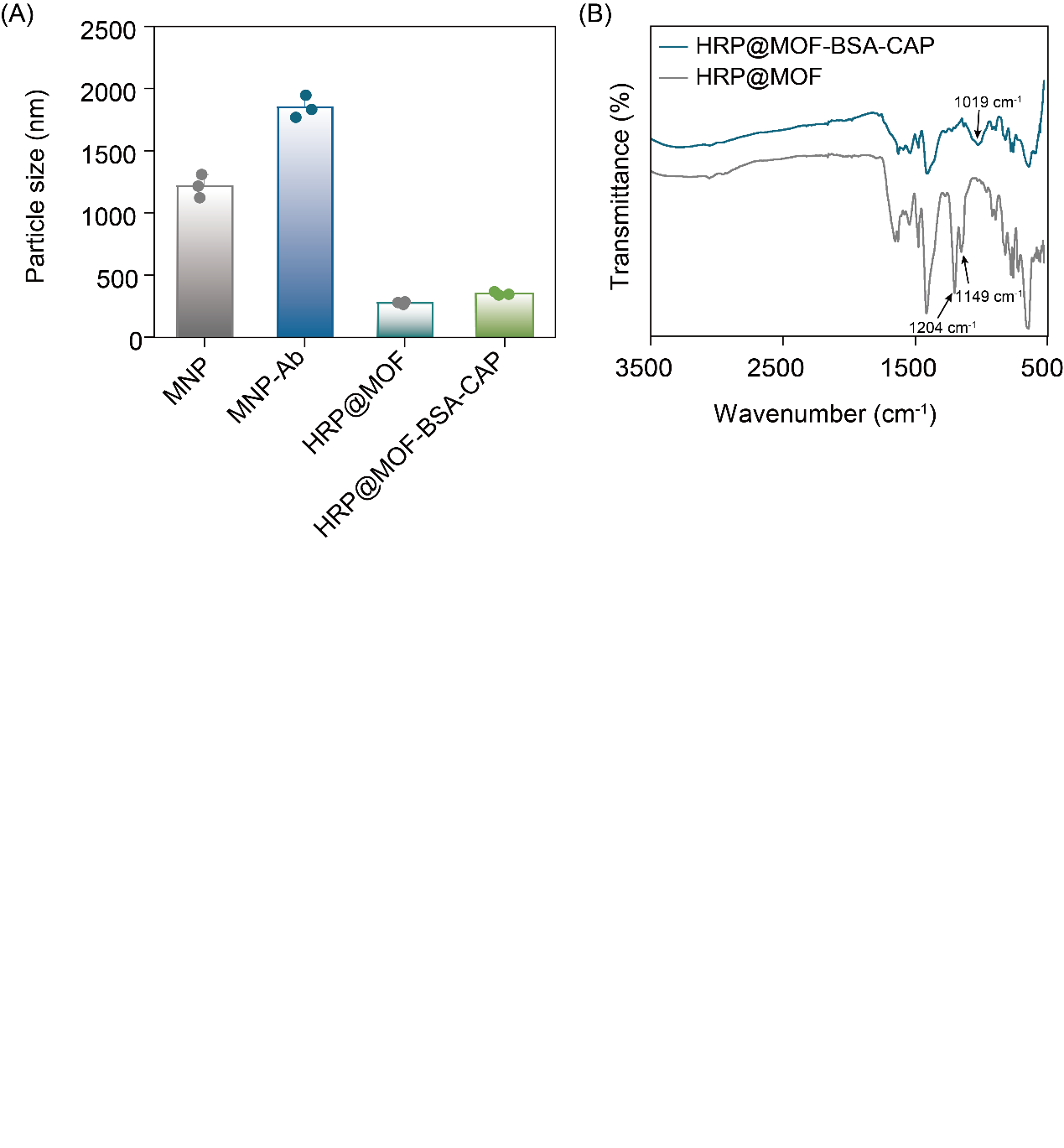


**Figure S3.** (A) Particle size of different materials, (B) FT-IR spectra of HRP@MOF and HRP@MOF-BSA-CAP. (MOF: Fe-NU-1003)


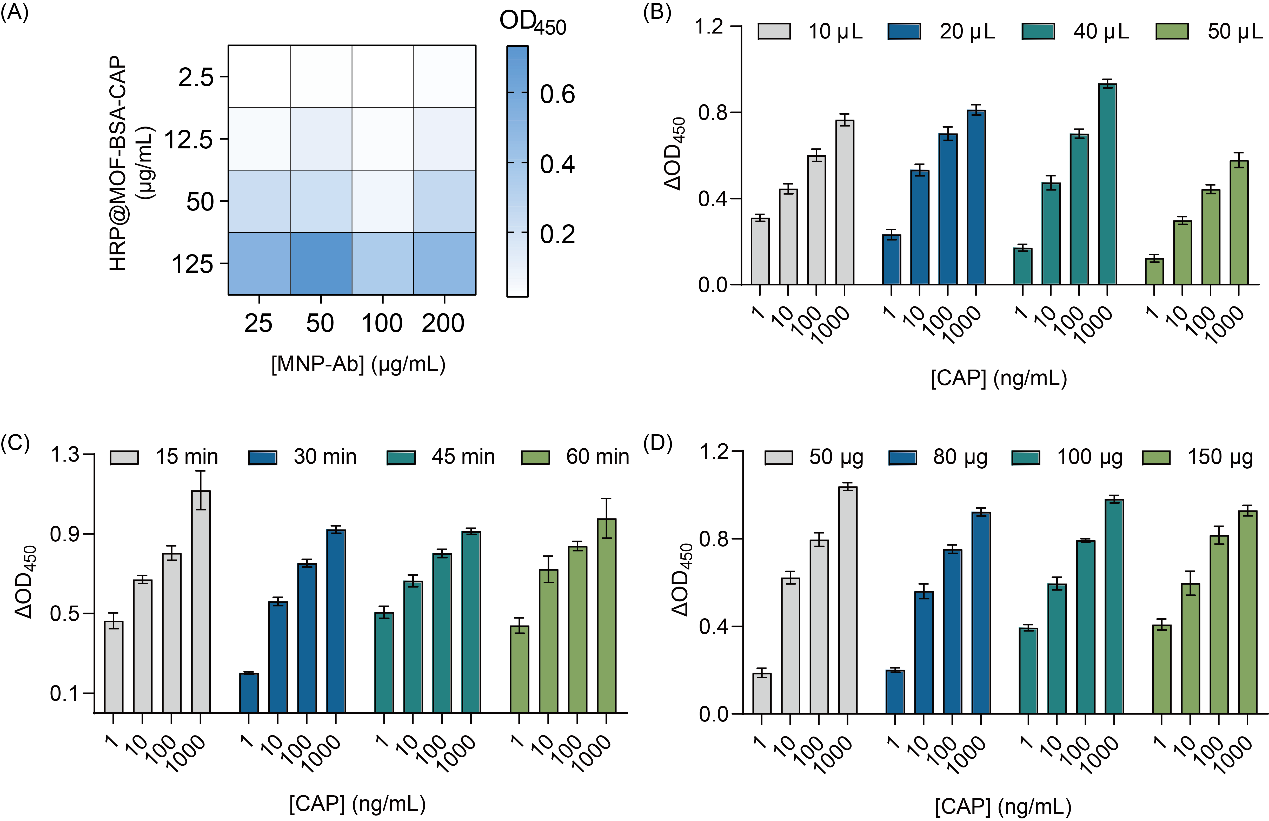


**Figure S4.** Optimization of conditions for detection (A) the ratio of the capture probe to the detection probe, (B) the dosage of coupling agent, (C) competitive reaction time, (D) the dosage of biometric molecules.


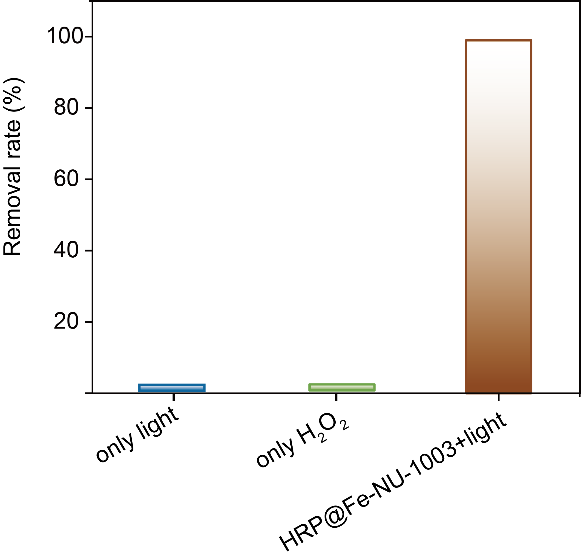


**Figure S5.** Control experiments for the CAP degradation.


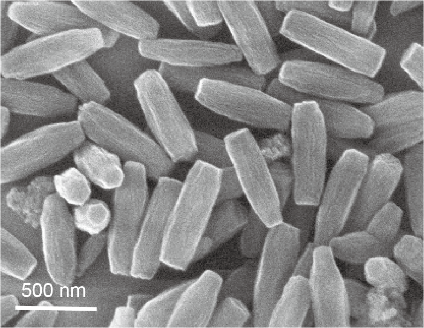


**Figure S5.** SEM images of HRP@Fe-NU-1003 after 5 cycles.


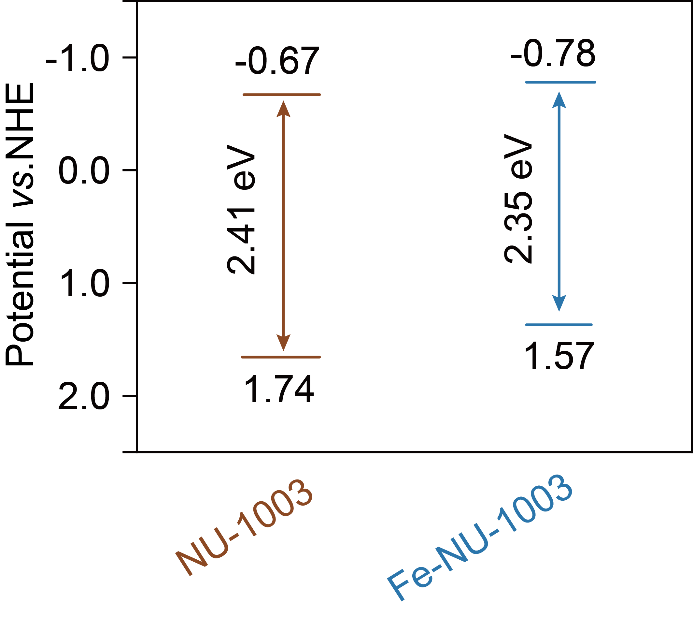


**Figure S6.** Band energy diagram of NU-1003 and Fe-NU-1003.


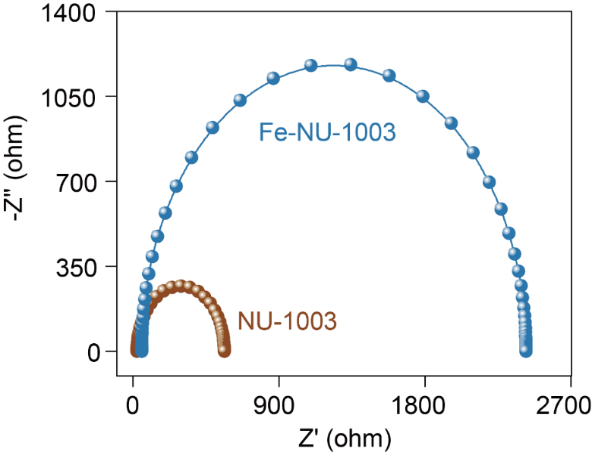


**Figure S7.** Electrochemical impedance spectroscopy (EIS) spectra of NU-1003 and Fe-NU-1003.


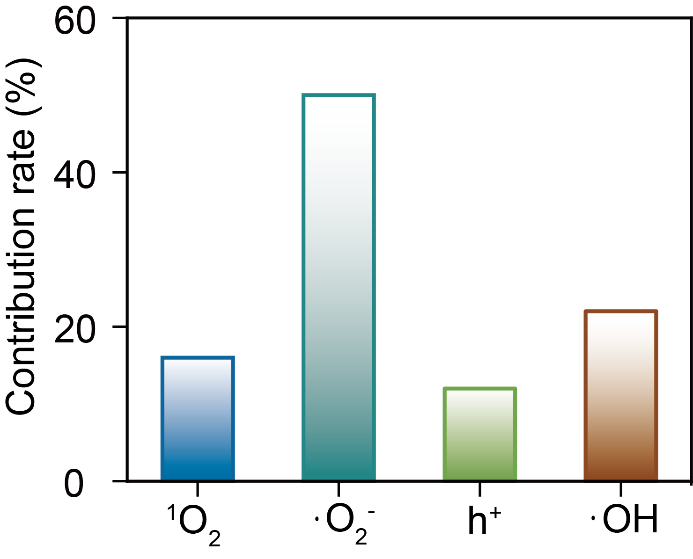


**Figure S8.** The contribution rates of different active species.

7. Supplementary Tables

**Table S1.** The CAP detection performance of different methods reported in literatures.

| Biosensor/Method | Time  (min) | LOD  (ng/mL) | Working range (ng/mL) | Reference |
| --- | --- | --- | --- | --- |
| ELISA^a)^ | 90 | 0.060 | 0.1-1 | ^[9]^ |
| Fluorescence | 30 | 14.210 | 646-25840 | ^[10]^ |
| Colorimetric | 330 | 0.003 | 0.001-1000 | ^[11]^ |
| DPV^c)^ | - | 16.150 | 1.615-32.3 | ^[12]^ |
| Fluorescence | 50 | 0.020 | 0.032-3.23 | ^[13]^ |
| LFIA^d)^ | 20 | 0.500 | 3-7 | ^[14]^ |
| CV^e)^ | 90 | 43.313 | 61.37-132701.32 | ^[15]^ |
| Microfluidic chips | 20 | 0.050 | 0.5-10 | ^[16]^ |
| SERS^f)^ | - | 3.230 | 3.23-323000 | ^[17]^ |
| HRP@MOF | 35 | 0.015 | 0.1-100 | This study |

**Table S2.** Spiked recoveries of CAP in real samples (n=3).

| Food matrix | Spiked  (ng/mL) | Detected  (ng/mL) | Recovery (%) | RSD (n=3)  (%) |
| --- | --- | --- | --- | --- |
| Fish | 20 | 18.40 | 92.00 | 4.16 |
|  | 200 | 172. 80 | 86.40 | 1.54 |
|  | 1000 | 1041.21 | 104.12 | 1.51 |
|  | 10000 | 10076.78 | 100.77 | 2.05 |
|  | 100000 | 100789.50 | 100.79 | 1.78 |
| Farm Wastewater | 20 | 22.51 | 112.53 | 11.85 |
|  | 200 | 213.17 | 106.59 | 16.72 |
|  | 1000 | 1318.24 | 131.82 | 4.14 |
|  | 10000 | 9563.87 | 95.64 | 10.08 |
|  | 100000 | 127198.13 | 127.20 | 12.24 |
| Urine | 20 | 18.89 | 94.43 | 16.23 |
|  | 200 | 200.68 | 100.34 | 6.77 |
|  | 1000 | 1032.02 | 103.20 | 6.03 |
|  | 10000 | 9043.67 | 90.44 | 8.98 |
|  | 100000 | 107232.50 | 107.23 | 6.46 |

**Table S3.** Real sample testing for chloramphenicol detection (n=3).

| Sample | Method | 1 | 2 | 3 | 4 | 5 | 6 | 7 | 8 | 9 | 10 | 11 | 12 |
| --- | --- | --- | --- | --- | --- | --- | --- | --- | --- | --- | --- | --- | --- |
| Fish | This method | 0 | 0 | 1293.00±53.16 | 0 | 0 | 0 | 100.76±  12.28 | 0 | 0 | 0 | 328.45±  9.60 | 0 |
|  | ELISA | 0 | 0 | 1439.65±58.74 | 0 | 0 | 0 | 0 | 0 | 0 | 0 | 312.88±  8.43 | 0 |
|  | HPLC-MS | 0 | 0 | 1241.60±43.89 | 0 | 0 | 0 | 105.43±  9.35 | 0 | 0 | 0 | 326.701±9.54 | 0 |
| Urine | This method | 0 | 102.17±  7.91 | 0 | 0 | 477.91±  43.25 | 0 | 0 | 0 | 1326.16±85.22 | 0 | 0 | 0 |
|  | ELISA | 0 | 0 | 0 | 0 | 0 | 0 | 0 | 0 | 1403.33±105.14 | 0 | 0 | 0 |
|  | HPLC-MS | 0 | 110.80±  8.34 | 0 | 0 | 493.85±  46.50 | 0 | 0 | 0 | 1259.03±75.38 | 0 | 0 | 0 |
| Waste water | This method | 1174.96±53.13 | 0 | 0 | 0 | 518.10±  34.03 | 0 | 0 | 0 | 0 | 0 | 0 | 2607.22±82.91 |
|  | ELISA | 1240.55±67.22 | 0 | 0 | 0 | 0 | 0 | 0 | 0 | 0 | 0 | 0 | 2550.69±96.65 |
|  | HPLC-MS | 1146.29±62.66 | 0 | 0 | 0 | 513.45±  27.69 | 0 | 0 | 0 | 0 | 0 | 0 | 2679.40±75.66 |

**Table S4.** The isothermal parameters of HRP@Fe-NU-1003.

| **Sorbent** |  | **Langmuir isotherm** |  | **Freundlich isotherm** | |
| --- | --- | --- | --- | --- | --- |
|  |  | **q_m_ (mg/g) K_L_ (L/mg) R^2^** | | **K_F_ (mg^1-n^L^n^g^-1^) 1/n R^2^** | |
| **HRP@Fe-NU-1003** |  | 545.66 0.012 0.988 | | | 38.71 0.43 0.979 |
|  |  |  |  |  |  |

**Table S5.** The concentration of H_2_O_2_ during the reaction process.

| Entry | Time (min) | H_2_O_2_ (ug/mL) |
| --- | --- | --- |
| 1 | 0 | 0 |
| 2 | 10 | 5.9 |
| 3 | 20 | 9.5 |
| 4 | 30 | 17.1 |

8. References

[1] P. Li, S.-Y. Moon, M. A. Guelta, L. Lin, D. A. Gómez-Gualdrón, R. Q. Snurr, S. P. Harvey, J. T. Hupp, O. K. Farha, *ACS Nano* **2016**, *10*, 9174-9182.

[2] X. Liao, X. Zhang, W. Wang, C. Liu, W. Yang, D. J. B. Wang, *Biosens. Bioelectron.* **2023**, 220, 114906

[3] M. J. Frisch, G. W. Trucks, H. B. Schlegel, G. E. Scuseria, M. A. Robb, J. R. Cheeseman, G. Scalmani, V. Barone, B. Mennucci, G. A. Petersson, H. Nakatsuji, M. Caricato, X. Li, H.P. Hratchian, A. F. Izmaylov, J. Bloino, G. Zheng, J. L. Sonnenberg, M. Hada, M. Ehara, K. Toyota, R. Fukuda, J. Hasegawa, M. Ishida, T. Nakajima, Y. Honda, O. Kitao, H. Nakai, T. Vreven, J. A. Montgomery, J. J. E. Peralta, F. Ogliaro, M. Bearpark, J. J. Heyd, E. Brothers, K. N. Kudin, V. N. Taroverov, T. Keith, R. Kobayashi, J. Normand, K. Raghavachari, A. Rendell, J. C. Burant, S. S. Iyengar, J. Tomasi, M. Cossi, N. Rega, J. M. Millam, M. Klene, J. E. Knox, J. B. Cross, V. Bakken, C. Adamo, J. Jaramillo, R. Gomperts, R. E. Stratmann, O. Yazyev, A. J. Austin, R. Cammi, C. Pomelli, J. W. Ochterski, R. L. Martin, K. Morokuma, V. G. Zakrzewski, G. A. Voth, P. Salvador, J. J. Dannenberg, S. Dapprich, A. D. Daniels, O. Farkas, J. B. Foresman, J. V. Ortiz, J. Cioslowski, D. J. Fox, Gaussian 09 (Revision D.01), I. Gaussian, Wallingford, CT, **2013**.

[4] R. Krishnan, J. S.Binkley, R. Seeger, J. A. Pople, *J. Chem. Phys.* **1980**, *72*, 650-654.

[5] F. Weigend, R. Ahlrichs, *Phys. Chem. Chem. Phys.* **2005**, *7*, 3297-3305.

[6] S. Xu, T. He, J. Li, Z. Huang, C. Hu, *Appl. Catal. B: Environ.* **2021**, *292*, 120145.

[7] S. Grimme, J. Antony, S. Ehrlich, H. Krieg, *J. Chem. Phys.* **2010**, *132*, 154104.

[8] F. Weigend, *Phys. Chem. Chem. Phys.* **2006**, *8*, 1057-1065.

[9] C. Liu, D. Deng, D. Xu, K. Wu, H. Yang, K. Zhao, J. Li, A. Deng, *Anal. Methods-UK.* **2019**, *11*, 507-516.

[10] Q. Wang, X. Qi, H. Chen, J. Li, M. Yang, J. Liu, K. Sun, Z. Li, G. Deng, *Microchim. Acta.* **2022**, *189*, 272.

[11] C. Yan, J. Zhang, L. Yao, F. Xue, J. Lu, B. Li, W. Chen, *Food Chem.* **2018**, *260*, 208-212.

[12] W. Yi, Z. Li, C. Dong, H.-W. Li, J. Li, *Microchem. J.* **2019**, *148*, 774-783.

[13] P. Ma, H. Guo, N. Duan, X. Ma, L. Yue, Q. Gu, Z. Wang, *Talanta* **2021**, *230*, 122349.

[14] S. Wang, H. Wang, T. Du, T. Bu, J. Xu, S. Liu, X. Yin, Y. Wang, D. Zhang, J. Sun, *Food Chem.* **2022**, *393*, 133351.

[15] S. V. Selvi, N. Nataraj, *Microchem. J.* **2020**, *159*, 105580.

[16] M. Zhao, X. Li, Y. Zhang, Y. Wang, B. Wang, L. Zheng, D. Zhang, S. Zhuang, *Food Chem.* **2021**, *339*, 127857.

[17] Y. Ding, X. Zhang, H. Yin, Q. Meng, Y. Zhao, L. Liu, Z. Wu, H. Xu, *Sensors* **2017**, *17*, 2962.
